# “*At what line does poor horsemanship cross the line to abuse, for you?”* Social media discourse around high-profile incidents in the performance horse world reveals polarised views on animal welfare

**DOI:** 10.1017/awf.2026.10093

**Published:** 2026-07-15

**Authors:** Erica Cheung, Daniel Mills, Beth Ann Ventura

**Affiliations:** 1 Department of Life Sciences, University of Lincoln – Brayford Campus, Lincoln, UK; 2 College of Veterinary Medicine, Department of Large Animal Clinical Sciences, Michigan State University, East Lansing, MI, USA

**Keywords:** Animal welfare, moral disengagement, performance horses, sociocultural barriers, speciesism, tribalism

## Abstract

High profile equestrians set an example for equestrian culture as well as public perception of horse sports. The present instrumental case study considers two incidents shared across social media. One is a dressage session featuring an elite equestrian striking at a horse 24 times with a long whip during a two-minute video. The second scenario shows a high-profile rider whipping and spurring a horse for a jump refusal during an international showjumping competition. We collected (and anonymised) 416 social media responses from a performance horse welfare equestrian group, general horse welfare advocacy groups, and public comments on the case videos. Using discourse analysis, we aimed to examine: (1) how individuals respond to the potentially welfare-compromising actions of high-profile equestrians; (2) what the responses tell us about people’s attitudes towards performance horses and their welfare; and (3) what we can learn about the social construction of animal welfare. Two polarised discourses encompassing the topics of the equestrians, the horse, the practice, and the industry are described. Findings demonstrate how enculturated practices and attitudes may prompt moral disengagement from potential horse welfare issues. We suggest that tribalism between those who support the horse training practices observed and other members of society may underpin the observed polarisation of views on horse welfare. Furthermore, this case study provides insight into the social construction of animal welfare in a social media setting. Consideration of these findings brings important insight into the sociocultural barriers and opportunities for the promotion of improved animal welfare.

## Introduction

Horse welfare in the context of equestrian sports has become an increasingly contentious topic, exacerbated by public concerns for the care and training of performance horses elicited by publicised incidents. Social media rapidly exposes potential horse welfare violations in the performance horse industry to a broad audience, including the public as well as other equestrians. It is also a medium that can encourage immediate and emotional responses to issues (Tucker *et al.* 2017; Hyvärinen & Beck 2018; Wahl-Jorgenson 2018; Chung & Zeng 2020). Unsurprisingly, animal welfare topics can provoke strong emotional responses on social media (Riggio *et al.* 2023). In recent years, social media discourse has become an important source for social scientists in revealing attitudes and perspectives on important social issues, such as climate change, immigration policy, and politics (Bouvier 2015; Ekman 2019; Krzyżanowski & Tucker 2018). Since animal welfare is considered a matter of public interest (Cowen 2006; Lusk & Norwood 2008; Lusk & Norwood 2011; Johansson-Stenman 2018; Espinosa & Treich 2024), it is not surprising to see horse welfare issues as a topic of debate in social media discourse. Therefore, social media discourse in response to animal welfare issues, including those in the performance horse industry, may be relevant to understanding the attitudes (i.e. the largely consistent evaluation of any given subject or object [Stangor 2020]) of interested groups in a social context.

More broadly, social media discourse may be a venue where animal welfare is *socially constructed* (Muhammed *et al.* 2022; Gilbert *et al.* 2025) as commentators share sociomoral evaluations on what they observe. Sociomoral evaluations are opinions grounded in the shared social norms and morals relevant to an individual’s cultural context (Youniss 1981; Berger & Luckmann 2011). Thus, individuals outside of the equestrian industry may hold perspectives on horse welfare that differ from those within the industry based on their own social and cultural background. This is particularly relevant to the conceptualisation of animal welfare, which can be conceived in a multitude of ways (Fraser *et al.* 1997; Weary & Robbins 2019; Muhammed *et al.* 2022). Likewise, members of the equestrian industry may share their perspectives in response to horse welfare issues exposed on social media.

In previous work, we found that participants in classical equestrianism are aware of and concerned about horse welfare but may be hindered from confronting certain welfare issues due in part to enculturation (Cheung *et al.* 2025). Enculturation is a socially facilitated process whereby individuals adopt the practices and attitudes of others (Mead 1963), leading to the maintenance of cultural norms (Herskovits 1949; Shimahara 1970) and creating potential blind spots to alternative views (Roth 2001). Input from those outside the enculturated group might then be required to drive cultural change. However, this is not a simple matter, as social group dynamics often cause resistance to outsider influence (Hornsey & Esposo 2009). To our knowledge, the interaction of insider and outsider views on social media in the context of equestrian practices has not been studied previously.

The present study evaluates the discourse on social media surrounding two incidents that took place in 2024 involving highly successful equestrians. These two events form an instrumental case study, which allows analysis of responses to potential horse welfare issues across more than one context (Stake 1995, 2005; Maréchal *et al.* 2010; Denzin & Lincoln 2011). The purpose of this study was to answer the questions: (1) how do individuals respond to the potentially welfare-compromising actions of high-profile equestrians? (2) what do the social media responses to the cases presented tell us about people’s attitudes towards performance horses and their welfare? and (3) What can we learn from this case study about the social construction of animal welfare? We use qualitative discourse analysis to evaluate public responses to these two incidents broadcast on social media. Studying discourse in this context is important because it reveals how equestrian cultural norms may be maintained or challenged by people when presented with an issue publicised as an animal welfare concern. More broadly, this approach provides insight into how issues of moral concern in animal welfare are addressed in a social environment.

## Materials and methods

### Ethical statement

This research was approved by the University of Lincoln Ethics Committee (ethics reference # UofL2024_18876 and UofL2025_18876).

### Case descriptions and rationale for selection

This instrumental case study consists of two performance horse scenarios (Cases A and B, below) captured on video in the competitive disciplines of dressage and showjumping. Instrumental case study facilitates the understanding of phenomena occurring within the case(s) being examined rather than being focused on the cases themselves (Stake 2005; Denzin & Lincoln 2011). We selected these cases because (a) they capture the potentially welfare-compromising actions of high profile, highly respected equestrians, and (b) a breadth of social media responses could be gathered for analysis. Further details of the individuals involved and links to the videos for the cases are not provided here due to ethical considerations involving the protection of the human case subjects (a requirement of our ethical approval).

Case A is a training scenario recorded four years prior to its widely shared public release on several social media platforms in 2024. The 2-min video shows a horse being ridden in an indoor arena and an internationally decorated dressage champion on the ground providing instruction. This individual is seen following the horse through the arena whilst holding a long lunge whip and striking at the horse’s legs at frequent intervals. At points in the video, the person strikes at the horse holding the whip in two hands using an overhanded motion. The horse was struck at 24 times within the 2-min time interval of the recorded session. The horse exhibits several behavioural signs of avoidance and distress, including evasion of the whip, kicking, and tail swishing. An individual off camera is heard laughing when the horse first displays these behaviours. On the day that the video became available online, the dressage champion released a public statement expressing regret for an “error of judgement” displayed in the video and stated that their actions were “out of character”. They went on to express shame and apologised for letting down their team, the public, and sponsors, but did not directly mention horse welfare nor concern for the horse.

Case B features another decorated equestrian, a showjumping champion from a different continent, riding a horse in a high-level competition at a prestigious showground in 2024. The video was not distributed on social media until 2025. In the video, the horse refuses to move forward and evades the bit as the rider attempts to manoeuvre the horse into the show ring. Eventually, the horse proceeds through a course of 1.30-m fences before slowing and then stopping at a fence. The horse is turned around sharply by the rider to reattempt the jump, with the rider spurring the horse as they approach the fence and striking the horse on the flank in mid-air over the jump. Upon landing, the horse balks at going forward, at which point the rider whips the horse four times in succession on the flank while spurring the horse and holding the reins tightly as the horse spins and appears to try to avoid going forward. When the horse stops moving, the rider gives a pat on the neck and proceeds out of the ring. Following report of the incident to a national equestrian sport governing body, the rider was represented by a spokesperson in a statement published in horse sport media. The statement confirmed that the rider’s actions had not been deemed a horse welfare violation by sport governing bodies, that the rider loves horses, and they would continue to ensure their welfare and safety. They went on to say that the rider responded to the horse’s behaviour in the moment but, in hindsight, the rider felt they should have taken a different approach.

Similarities and differences between Case A and B are important to note. Case A was widely characterised by news media and equestrian governing bodies as a horse welfare violation, with the individual featured in Case A receiving disciplinary action by relevant governing bodies and being dropped by brand sponsors. In contrast, Case B was not considered a horse welfare violation by equestrian sport governing bodies, the rider received no disciplinary action, nor was there overt wider media coverage of the issue. Case A occurred at a private venue, while Case B occurred at a competition open to the public. In both cases, the human subjects are women.

#### Data collection

Case study data consisted of comments made in response to the case videos posted and shared on the social media platforms Facebook and YouTube. We collected data from publicly available groups and pages that were indicated as allowing public access and where the original post was viewable without requiring login or permission to view. Data were collected between 13 August–30 September, 2024 for Case A and between 11 April–1 May, 2025 for Case B. Data collection for each case concluded when it appeared that no new comments were being made on consecutive days.

The social media groups where data were collected included both public and equestrian-specific forums. Public forums were considered those that did not specify a particular identity within equestrian sport, while equestrian forums were those that required members to be equestrians to comment. We did not seek to identify commentator demographics for reasons of respondent protection. Where possible and relevant, we characterised comments as “non-equestrian” and “equestrian”. “Non-equestrian” comments were defined as those from individuals who were not explicitly or implicitly identified as equestrians or who self-identified as non-equestrian within their comments. “Equestrian” comments were those from individuals who self-identified in their commentary as such, or from those within equestrian-specific social media groups. Any personal identification, including social media handle, name, geographic locations or other identifiers, was removed from the data prior to coding and analysis to preserve commentator anonymity.

All comments initially collected were included in the final dataset. Comment length ranged from a single word to several paragraphs. For Case A, the data consisted of 39 comments gathered from one Facebook group for equestrians dedicated to performance horse welfare, and 200 comments made in response to the video on YouTube. Data from Case B included 46 comments from one Facebook group for equestrians dedicated to performance horse welfare, and 131 from two Facebook groups focused on horse welfare advocacy (as this video had not been posted to YouTube at the time of this study). This resulted in a total of 416 comments spanning the two cases.

#### Data analysis

Qualitative discourse analysis was utilised for this work. This methodology conceptualises discourse as a window of insight into how the world is constructed, represented and understood by individuals in a social context (Gill 2000; Potter &Wetherell 2002; Burr & Dick 2017). Discourse is operationally defined as any written or verbal communication (Gill 2000). In this study, discourse consists of the social media comments collected. Discourses contribute to the development and maintenance of concepts in a social context (Jorgensen & Phillips 2002) and are also a way of identifying with one’s social group (van Dijk 1997; Eriksson & Kovalainen 2008).

Discourses are analysed by critically observing the construction of the discourse as well as its role in constructing meaning in social contexts (Potter & Wetherell 2002; Gill 2000; Gee 2014). Therefore, the study was situated in a social constructivist paradigm, which holds the ontology that truth is relative to each person’s perspective and that reality is socially constructed through interactions with others (Guba & Lincoln 1994; Searle 1995; Yin 2003). The epistemology of this paradigm is an interpretivist one wherein the interaction of the researcher with the discourse contributes to the interpretation of meaning from it (Guba & Lincoln 1994; Creswell & Poth 2016). Importantly, we did not seek to interpret individuals’ perspectives during the analysis, nor did we utilise a pre-determined analytical framework to interpret the discourse. In keeping with the goal of discourse analysis (Gill 2000; Potter & Wetherell 2002; Gee 2014), we focused on how the discourses contributed to the construction of hypotheses about how people may broadly perceive horses and conceive of their welfare.

An inductive open coding approach driven by the data was used during the iterative process of coding and analysis. Coding was conducted manually (without the use of coding software) by EC after familiarisation with the data and initial notes on evident patterns. Descriptive codes were used without any deductive analytical framework in mind to inform the clustering of the discourses and the formation of categories of broader meaning (Gee 2014). For example: where we interpreted individuals’ comments as derogatory towards or protective of the human subjects of Case A and B, we coded these as “*ad hominem attack”* and “*in defence”*, respectively. These codes contributed to the discourse topic of *“the equestrians”*, which led to further discussion of the function of this discourse later on. Each social media comment was evaluated individually, followed by comparison across the dataset of both cases. When coding, we considered the vocabulary used, such as particular words or phrases, and rhetorical strategies individuals employed, such as emotional metaphors, in their responses (Gill 2000; Jerit 2008; Gee 2014). This was considered in the greater context of the discourse as a whole as it related to people’s attitudes towards horses and how they contend with potential horse welfare issues and common equestrian practices. Research findings are illustrated by the inclusion of quotes from the social media comments; these have been either truncated to reduce traceability or subjected to a Google search to confirm the originating source cannot be readily found. Quotes are denoted as either CA or CB to indicate whether they were derived from Case A or Case B, respectively.

#### Positionality of the researchers

Qualitative discourse analysis is influenced by the positionality of the researcher and transparency is key to the robustness of the research; positionality statements thus offer context on the experiences and beliefs researchers bring with them to the construction of knowledge (Gee 2014; Creswell & Poth 2016; Braun & Clark 2022). EC comes to this work as a horse owner with experience in equestrian sports; she holds a post-graduate degree in animal behaviour and welfare. This study forms part of her PhD degree focused on horse welfare. She holds a personal position on animal welfare aligned with the Five Domains model (Mellor *et al.* 2020). From this perspective, EC perceived Case A and B to represent horse welfare violations, and this influenced the framing of discussion in this work.

DM is an internationally recognised authority on companion animal, including equine, behaviour. He holds a PhD in equine behaviour and welfare and is Professor of veterinary behavioural medicine at the University of Lincoln, where he is the lead of the Animal Behaviour, Cognition and Welfare Research Group. He has never engaged in any competitive equestrian sports. He has developed the psychobiological approach to assessing the internal motivational and emotional state of animals in a clinical setting, which is also applied to assessing their well-being (Mills 2025). BV holds a PhD in animal welfare and is an associate professor of animal welfare within the College of Veterinary Medicine at Michigan State University. She has never owned horses nor competed in equestrian sports but has long enjoyed being around and working with them in a range of contexts throughout her life and career.

## Results

**Figure 1.**
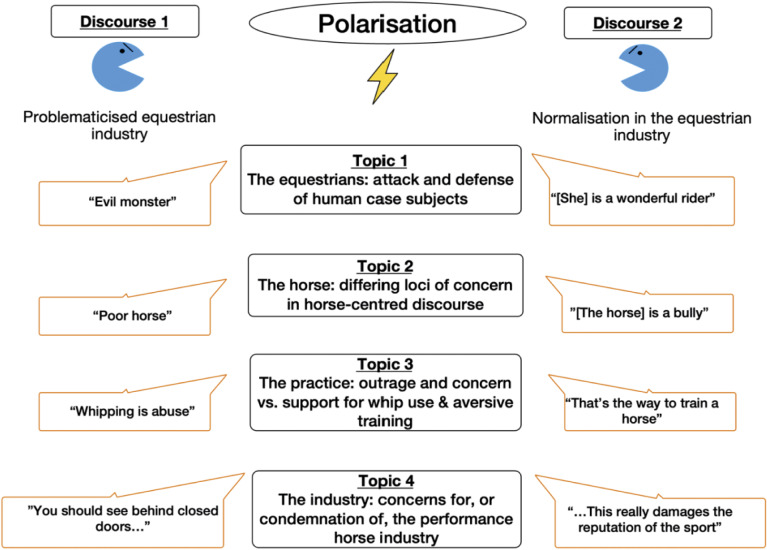
Discourse analyses of (n = 416) social media comments on two potential horse welfare violations were conducted. Two polarised discourses (Discourse 1 and Discourse 2) were interpreted including four topics illustrated here.

We interpreted two parallel discourses from social media comments, wherein there appeared to be two polarised perspectives framing the responses to the incidents depicted in Cases A and B. We describe *Discourse 1* as that of those who were critical of equestrians, equestrian practices, the equestrian industry and expressed explicit concern for the horse. This group also appeared to perceive the incidents of Case A and B to be a welfare issue. In contrast to *Discourse 1*, in *Discourse 2* we observed criticism of the outrage expressed by others and defence of equestrians and equestrian practices. Rather than concern for the horse, this stream of discourse focused concern on how moral outrage may detrimentally affect the reputation of the equestrian industry. Within *Discourse 2*, an alternative perspective in judging the events of the cases was presented where abuse was differentiated from poor horsemanship. Both discourses were directed in focus toward one (or more) of the following topics: (1) the equestrian; (2) the horse; (3) the practice; and (4) the equestrian industry. We use quotes from each of the opposing discourses to illustrate the representations made at each level. Figure 1 provides an overview of the findings.

### Topic 1: *The equestrians* – attack and defence of the human case subjects

First, we explore discourse that reveals attitudes towards the human case subjects, i.e. the trainer or rider in the videos. This consisted of *ad hominem* attacks in the *Discourse 1* stream, and *defence* of the human case subjects in *Discourse 2.*

#### Discourse 1: “Evil monster”

Philosophers define the *ad hominem* as a direct attack on a person’s *ethos*, or character, with intention to personally discredit them (Engel & Philosophy Documentation Center 1994). The *abusive ad hominem* goes further to threaten harm or insult particular traits of the person (Walton 1998). In the discourse analysed in this case study, we observed a focus on expressing varied levels of disdain for the equestrians of Case A and B.

Keywords in this discourse included description of the human case subjects themselves as “*stupid cruel girl*” (CA), “*evil monster*” (CA), “*horrible person”* (CB), and “*abuser”* (CB). Notably, the subjects in both cases were often referred to as a “*bitch”.* Both professional and personal sanctions were called for against the human subjects; these ranged from brief statements such as “*ban her for life”* (CA) to violent calls, “I would snatch the whip out of that b*tch’s hand and stitch her up so that even her own mother wouldn’t recognize her” (CB).

These *ad hominem* attacks were made by individuals in the welfare-advocacy-focused social media groups and, to a lesser extent, public social media groups. Within the exclusively performance-equestrian welfare group, there was no *ad hominem* discourse.

#### Discourse 2: “[She] is a wonderful rider”

Responses to the attacks toward the equestrians in Cases A and B occurred within *Discourse 2*, ranging from cautious support to strong defence of the equestrians. Among those who were more careful with their approach was one person who wrote*, “I’m not saying the trainer wasn’t wrong. That she withdrew, apologized and feels shame says a lot. When we know better, we do better”* (CA). Responding to several *ad hominem* comments, one individual in a non-equestrian group expressed empathy with the equestrian, sharing *“While I do not endorse her actions, it is crucial that she receives appropriate care and support…”* (CA). Others were more strident in their defence of the human subjects, especially when there were consecutive and severe threats directed towards the equestrians. One person, for example, responded to a threat to the credibility of the equestrian in Case B: *“STOP THIS NONSENSE TALK. [SHE] IS A WONDERFUL RIDER AROUND THE WORLD…”* they wrote in all uppercase, potentially emphasising the emphatic nature of their feelings. Such emotionally salient responses suggest that the criticism of the equestrians was potentially perceived as attacks on the wider equestrian community as well. Through the defence in these comments, they also demonstrate how people may be willing to overlook actions that they may not support when enacted by an otherwise highly regarded individual.

### Topic 2: *The horse* – differing loci of concern in horse-centred discourse

Here, individuals directed their focus to the horses in the Case A and B videos. Within *Discourse 1* this concern for the horse manifested as empathy for the horse’s experience. Conversely, in *Discourse 2* horse-centred comments appeared to direct blame toward the horses for eliciting the actions depicted in the video, rather than direct concern toward the subjective experience of the horses.

#### Discourse 1: “Poor horse”

Comments evidencing concern for the horse’s experience consisted of individuals’ assessments of the horses in the case videos. There were various iterations of *“poor horse”* in regard to Case A in the public social media group, suggesting that commentators may have empathic feelings towards the horse being whipped. Interestingly, we did not find any horse-focused discourse indicative of concern for the horse in Case A in the performance horse equestrian-specific group. In Case B, there were more nuanced remarks about the horse in the video: one person interpreted the scene as “*a horse clearly upset and overwhelmed”* (CB). Others thought the horse was “*anxious*” and “*afraid*” (CB). These remarks were made in the public or welfare advocacy groups. It is important to note that the comments expressing concern for the horses’ experience were not topics of dialogue between commentators. Rather, the commentary focused on statements that the horse was a victim.

#### Discourse 2: The horse is “a bully”

In contrast to *Discourse 1*, individuals contributing to *Discourse 2* appeared to perceive the horses in Case A and B as deliberately or consciously working against the human subjects, rather than as victims of human action. One person described the horse in Case A as *“deeply stubborn and obstinate* [*…*] *knows it has an inexperienced rider and is taking advantage”.* Similarly, the horse in Case B was called *“a bully* [*…*] *trying to see if she can intimidate the rider”* (CB). Others concluded that *“Some horses learn the easy way, and some just don’t. Horses are smart creatures, and they can learn to just refuse to do as they’re told*” (CB). This line of discourse demonstrates an anthropocentric perspective (that emphasises the cognitive underpinnings) of motivations for a horse’s behaviour.

Another aspect was how individuals appeared to perceive the effect of whipping on the horses. *“People have gone very soft, this is not abuse, it’s training, it does not hurt”* (CA) was one statement. In agreement, another replied, *“It doesn’t amount to abuse to me, horse’s movements aren’t affected and I don’t see any injury being caused”* (CA). These evaluations across the discourse suggest that individuals may differentiate actions intended for training from those with harmful intent or lasting harmful impact. Furthermore, the focus on physical pain or injury indicates a bias towards the physical aspects of well-being and perhaps a deprioritisation of the mental or affective domain of animal welfare. This is emphasised by statements indicating that the whipping was not abusive since it was not deemed physically injurious.

Some commenters appealed to ethological principles relating to equine social behaviour in a potential attempt to frame the horse’s experience of being whipped as natural: *“In the wild, horses live by strict rules. Herd dynamics are enforced clearly, sometimes harshly-even fatally”*, wrote one equestrian (CB). A follow-up agreed, *“Horses by nature thrive on direction given by a respected leader. This is how the species evolved and survived. They are lost without such a leader, and most cannot be comfortable enough to enjoy life without one”* (CB). The way that the experience of the horse is framed in this discourse suggests a potential misunderstanding of equine social behaviour or misuse of equine ethology.

### Topic 3: *The practice* – outrage and concern vs support for whip use and aversive training

Individuals responded in two contrasting ways to the whip use in Cases A and B. On one end of the spectrum was *Discourse 1*, where individuals expressed outrage and concern about training practices. This extended into commentary on when aversive training methods may be considered abuse. Opposite to this response was *Discourse 2*, in which individuals demonstrated support and justification for training practices employing aversive approaches.

#### Discourse 1: “Whipping is abuse”

There were expressions of outrage made toward the use of whips in both cases. This is exemplified in statements of judgement such as “*horrible whipping”* (CB), and *“disgusting show of violence”* (CA). Many comments more widely referenced whip use in general and as well as other training practices in equestrian sports, suggesting that whips, spurs, and bits should be minimised if not prohibited altogether. For example, one person in a welfare advocacy group asked, *“Why are we still using whips, bits and spurs on our ‘partners?’”* (CB).

Just as in the *ad hominem* discourse, outrage at whip use was noticeable early in the comment threads for both Case A and B and occurred primarily within the welfare advocacy groups and public forums. There were, however, a few comments in this discourse stream within the performance-equestrian specific group. *“When will we stop thinking it is ok to whip animals?”* one equestrian questioned amongst a series of comments supporting whip use in Case B. They continued with expression of concerns that people claim love for their horses whilst also supporting whip use and argued that *“whipping is beating”* (CB).

Judgement statements on the case scenarios concluded that *“whipping is abuse, full stop”* (CB), and that *“hitting a horse with intent is always abuse”* (CA). Such statements were made in response to the videos of Case A and B or directed at people that defended any aspect of the whipping incidents. Some individuals deliberated on how whipping should be judged. *“At what line does poor horsemanship cross the line to abuse, for you?”* one person asked of someone who disagreed with their decision that the whipping in Case A constituted abuse. However, there did not appear to be a conclusive dialogue on this matter.

#### Discourse 2: “That’s the way to train a horse”

Arguments within *Discourse 2* disputed the claims of those opposed to the practices or tools used in the videos. Potential reframing and trivialisation of the whip use in Case A and B appeared to be used to refute arguments that whipping constitutes abuse. Furthermore, individuals in *Discourse 2* referred to the events depicted in the videos as “poor horsemanship” rather than “abuse” when evaluating the impact of Case A and B.

“[*…*] *not abuse, just bad horsemanship”*, was one example of clarification on the definition of whip use as observed in Case B. Another example was a response to Case A, *“Not abusive. We have conflated abuse and poor horsemanship”.* This line of discourse between commentators about the perception of abuse and poor horsemanship resulted in criticism of those who were opposed to whip use: *“Your assumption of what is perceived as abuse is a false narrative”* (CB). This criticism also took the form of remarks on others’ perceived lack of knowledge about equestrian practices. *“Not one of you know or would understand the circumstances”* (CB), one person stated while another asked, *“When was the last time you trained a horse?”* (CB). A similar tone was detected in a retort directed at someone disagreeing with the horse training demonstrated in Case A, “*So you have no idea how to train a horse. That’s the way to train a horse”.* None of these comment threads progressed into constructive dialogue.

Potential trivialisation was also observed as a response to welfare concerns: *“This is not bad…and it seems not done in anger, she is only asking the horse to pick up its feet…not beating it”*, one person remarked (CA). Another questioned, “*Have you ever heard a raised voice to an athlete*?” and then explained, *“This is a brief moment of working into sustained suspension, and no blood was spilled* [*…*]*”* (CA). There were comments that appeared to trivialise whip use by contrasting human actions to equine social interactions in a herd setting: *“…a rider with a stick can’t compare to the kick or bite from another horse”* (CA).

Others justified whipping as necessary (thus trivialising its significance), as seen in a response to Case B where a person explained “napping”, an equestrian term for refusal to go forward: *“Just like teenagers*, [horses] *will test the rules to see if they truly apply”* and when horses figure out that napping gets them what they want “*they will [become] increasingly more dangerous in their behaviour”.* The commentator further justified whip use in that *“cooing and petting are likely what got it to this point in the first place”.* Repeatedly striking the horse in Case B was considered *“the acceptable fix for this* [*…*] *to get the horse going forward in any way possible* [*…*]*”* (CB). This sort of justification suggests that individuals may not see any alternative to whipping the horse, and this might serve to maintain normalised practices.

Whipping was similarly justified due to the perceived danger related to the horse’s behaviour of spinning and refusing to go forward. One person informed others that the horse was displaying *“DANGEROUS behavior”*and went on to say that “*Dangerous horses die”*, continuing that the actions such as the whip use displayed could “*save a difficult horse’s life”* (CB). An equestrian identifying as having experienced a riding injury agreed and expressed concern for the potential harm to the rider: *“Horses need to go forward* [*…*] *it’s for everyone’s safety”* (CB).

Reframing, in which an issue may be presented in a more positive light than initially perceived, was also observed in defence of whip use. For example, an equestrian responded to concerns expressed about Case A in the public social media group by offering an anthropocentric reframing: *“*[*…*] *a lot of things can appear “harsh” in horse training that truly are not. Yes, training is often uncomfortable for horses, but so are a lot of things in life for any person or creature”.* The argument that equine social behaviour was analogous to training practices occurred as a reframing of whip use: *“Horses kick and bite each other all the time to signal to one another to move over, etc.* […] *you can observe that they communicate with each other by aggressive touching all the time”* (CA). The metaphor of a parent-child relationship and parental discipline was an analogy seen as well across *Discourse 2* to reframe aversive training practices. For example, one person responded to Case B saying that *“A strict parent with loving intentions”* is better than a parent allowing any behaviour “*out of apathy”.* They went onto say that this applied to horse training where “*Discipline rooted in care and consistency builds trust”*, (CB).

### *Topic 4:* The industry –*condemnation of, or concerns for, the performance horse industry*

Concern about and even condemnation of the performance horse industry was observed in *Discourse 1* of this final topic. Within this discourse, the events of Case A and B seemed to initiate or exacerbate concerns that all industry members conduct themselves similarly. This perception appeared to lead to condemnation of horse sports in general by those in *Discourse 1.* Conversely, despite the defences mounted against critics in *Discourse 2*, there also appeared to be concern expressed for the performance horse industry by its supporters. In addition, commentators in *Discourse 2* seemed aware of the threat to public perception brought about by the incidents in Case A and B.

#### Discourse 1: “You should see behind closed doors…”

The behaviour of the equestrians in Case A and B was potentially perceived by observers as demonstrative of equestrianism as a whole. *“She’s a multi gold medalist top of her sport! If that’s what she does I’ll assume that’s what most do”* (CA) stated one person. Another similarly implied that the actions in the video were not an isolated incident, *“You should see behind closed doors in every equine discipline. This is nothing”* (CA). Those critical of the equestrian industry mentioned animal welfare in relation to horse sport governing bodies and how they *“don’t care about the welfare of the horses”* (CA) and raised the need for them to develop *“clearer guidelines, policies, and procedures concerning the best welfare for horses”* (CB). This indicates a shift in, or extension of, responsibility from participants within the industry to the organisations governing it, to develop and enforce stronger regulations.

This discourse included concern that using animals in sports was harmful in general. Statements expressing the highest level of concern suggested that *“this sport and other animal ‘sports’ shouldn’t exist* [*…*] *forcing the poor creatures into unnatural movements for your own amusement, looks like torture to me”* (CA). Potential rejection of equestrian sports because of the perceived harm to horses was found amongst the public social media commentary and welfare advocacy groups.

#### Discourse 2: “Non-horsey people seeing things like this really damages the reputation of the sport…”

In contrast to the concerns and condemnation seen in *Discourse 1*, the focus for *Discourse 2* was on protecting the performance horse industry. Particularly in the equestrian-specific social media groups, people appeared deeply worried about the effect of perceived welfare violations on outsider views of the industry. An example is a comment on how the publicity of Case A brought *“huge negativity to the equestrian world”* (CA). They went on to say, *“Non-horsey people seeing things like this really damages the reputation of the sport and everyone that participates in it”.* Such statements suggest that there is an awareness of the social unacceptability should outsiders witness practices like the whip use in Case A. Indeed, some comments expressed disapproval of the level of moral distress others exhibited and the negative attention this could draw. These individuals described others’ response to Case A and B as *“sensationalist reactions”* (CB) and *“public outcries of abuse”* (CA) that would lead to the end of the performance horse industry. “*The animal rights extremists are watching and gathering comments”*, warned one, *“It’s not smart to virtual* [sic] *signal across social media”* (CB). One equestrian responded to discussion of the incident in Case B with the caution, “*We have to be careful as an industry”* since condemning practices without nuanced conversation would result in the horse industry being taken away.

Amongst the discourse focused on the performance horse industry, there were suspicions that the videos of the scenarios had been shared in order to expose issues in equestrian sports that would discredit equestrianism: *“Do not tell me it’s for* [horse] *welfare that it is coming out now”* (CA) one commentator in the equestrian specific group stated. Similarly, others were concerned that the exposure of cases like these was a tactic used to destroy the reputation of the industry. Another comment offered a suggestion that discouraged the sharing of the video of Case B: *“*[…] *we must focus on education and elevation-not attacking others.”* This implies that some may recognise the practices displayed in the videos as being publicly unacceptable while possibly wishing to protect norms within equestrian culture.

## Discussion

The present work aimed to evaluate how individuals respond to a perceived performance horse welfare issue and what these responses might reveal about attitudes towards horses and their welfare. We also aimed to further our understanding of how animal welfare may be socially constructed. Through discourse analysis, we explored four topics within two parallel yet opposing discourses. There were contrasting perspectives shared and a variety of arguments made in criticism and defence of equestrians, horse training practices, and the equestrian industry. Commentators also made statements of their individual positions, criticised differing views, and appeared to have differing priorities in their focus of concern. It is worth noting here that whip use in equestrian activities is a traditional norm, but also a practice that has become more controversial in the public sphere and questioned as a threat to horse welfare by welfare scientists (World Horse Welfare 2024; Uldahl & Mellor 2025). This case study demonstrates one way in which animal welfare may come to be socially constructed through online media when individuals share their opinions and values. Furthermore, the evidence presented here highlights a polarisation of views on a perceived horse welfare issue.

The evident polarisation of ideas shared in the present study shows how social contexts such as equestrianism influence the construction of animal welfare and contribute to the complexity of solving welfare issues due to a lack of consensus on what constitutes “unnecessary suffering” (a legally defined term underpinning animal welfare legislation – e.g. the U.K.’s Animal Welfare Act 2006) or indeed even a harm to animal welfare. Here, we discuss the potential mechanisms underpinning the polarisation of views based on the discourse analysed, including tribalism and moral disengagement. Finally, the issue of polarisation of opinion in relation to animal welfare is discussed as a barrier to positive change and potential ways forward.

### Tribalism

To reveal the broader implications of the discourse examined, we contextualise the findings within the social psychology concept of tribalism developed out of evolutionary biology (Darwin 1981/1871; Boyd & Richerson 2005; Greene 2013). This is defined as the loyalty and attachment to one’s own social group (Ferrera 2015) and is considered by some as a fundamental human social trait (Wilson 2012; Clark *et al.* 2019). Tribalism exists at many levels of human organisation, ranging from genetic kinship and geographic culture to political party alliances (Clark *et al.* 2019). Highly fragmented social groups such as sports fans, professional organisations, and even consumers of product brands are considered to generate a form of neo-tribalism (Maffesoli & Maffesoli 1996; Cova & Cova 2001, 2002; Armstrong 2003) based primarily on a community with shared interests and sentiment. A study of the professional composition of the equine industry in France proposed that equestrianism is a form of tribalism in that members of equestrian culture become deeply entrenched in the practices and attitudes of their sport (Grefe & Pickel-Chevalier 2015).

We propose that the discourses analysed in this study reflect the outcome of enculturated equestrian practices and attitudes that come into conflict with the moral views of at least some social groups outside of equestrianism. The discourses interpreted might be, at least in part, the result of “tribalism” amongst equestrians and interested groups in broader society.

Notably, the *ad hominem* attacks directed towards the equestrians in Case A and B were observed only in the horse welfare advocacy and public social media groups, and not within the performance horse welfare group comprised of individuals identifying as equestrians. *Ad hominem* is considered a feature of tribalism as it is a manifestation of an “us vs them” attitude (Borovali 2018; Anthony 2020; Bradley & Roland 2022). Argumentative tactics that employ *ad hominem* are often the initial response to highly controversial or emotional issues triggering moral outrage (Walton 1998; Macagno 2013), but these hold little strength in persuasion (Rozin *et al.* 1999; van Eemeren *et al.* 2012). However, it does not appear that most individuals contributing to the discourse and utilising this argumentative tool intended to persuade others of their position. Therefore, it is proposed that the disparaging discourse analysed in this case study is rather a signal that moral transgression has been perceived (Rozin *et al.* 1999; Tetlock *et al.* 2000) and individuals choose to signal this to others to reinforce their tribal identity and associated moral positioning (Aquino & Reed 2002). According to the model of moral contagion developed by Brady *et al.* (2020), individuals share virtual messages of moral outrage on social media, and this effectively spreads to others who then come to similar moral evaluations and potentially form moral “tribes.”

Not only do *ad hominem* statements contribute to the socio-moral identity of a tribe, but they also contribute to its defence. In a discussion of argumentation in political debate, Borovali (2018) proposed that *ad hominem* arguments contribute to tribalism by focusing arguments on denigrating others of differing perspectives rather than promoting constructive discussion of diverse views. Thus, *ad hominem* attacks are potentially detrimental to the promotion of horse welfare within equestrian culture by creating resistance to change. The barrier to rational discourse caused by the *ad hominem* can be seen in the defensive responses in protection of equestrian culture and practice. The extent of loyalty and belonging to a group influences the willingness of individuals to overlook moral violations, especially when perpetrated by highly respected group members (Abrams *et al.* 2013; Travaglino *et al.* 2016). This could explain the discourse defending the equestrians in Case A and B, and the lack of decisive outrage by some towards the actions of those equestrians.

Further, we note that discourse (particularly *Discourse 2)* was highly anthropocentric. These findings suggest underlying speciesist attitudes possibly rooted in traditional equestrian views towards horses and their objectified value. Speciesism is defined as the bias in favour of one’s own species and the reduced moral value of some species over others (Singer 2002). Associated with speciesism is the trivialisation of animal emotion; those that consider humans as superior to non-human animals may be likely to trivialise the experiences of less cognitively sophisticated animals. This attitude has also been linked to negative perceptions towards less privileged social groups (Costello & Hodson 2010). It has been argued that tribalism is the root of speciesism (Machery 2016) as the tribalistic tendency to discriminate against those outside one’s social groups may extend to beings outside their species membership (Jaquet 2022).

### Moral disengagement

Moral disengagement is the cognitive process of rationalising one’s actions and attitudes when they conflict with one’s internal moral views to reduce moral distress (Bandura 1990). The process of moral disengagement can be motivated by tribalism, as individuals cognitively modify their moral concerns to maintain alignment with group norms and moral reputation as well as their self-identity within the group (Kelly 2013; Ashokkumar *et al.* 2019). This phenomenon has been studied in politics where strong partisan loyalty motivates people to ignore and justify behaviours that they would normally find reprehensible (Ashokkumar *et al.* 2019; Hull *et al.* 2024).

In the context of animal welfare, moral disengagement has been described predominantly regarding the meat paradox (i.e. the psychological dilemma raised by people’s love for animals and concern for animal welfare and their consumption of meat) (Joy 2009; Piazza *et al.* 2015; Bastian & Loughnan 2017; Loughnan & Davies 2020; Rothgerber 2020). More recently, Schüßler *et al.* (2024) demonstrated how media discourse surrounding the meat and dairy industry in Germany contributed to moral disengagement amongst consumers. Ultimately, the outcome of moral disengagement is reduced concern given to animal welfare and prioritisation of human interests.

Here, we posit that moral disengagement (Bandura 1990, 2002) might underpin the trivialisation, justification and reframing of whip use and attitudes in support of aversive training methods in Cases A and B. According to social cognitive theory, moral disengagement occurs when social group norms conflict with individual moral positions (Bandura 1990, 2002; Moore 2015). For example, an individual may support whip use as part of enculturated traditional equestrian practice yet find the broader concept relating to the unnecessary hitting of an animal as immoral.

Bandura’s moral disengagement theory (1990, 2002) is comprised of eight cognitive mechanisms. Below, we describe how these mechanisms may be at work in the discourse analysed in the present study:

1. Moral justification: justifying actions as socially valuable or moral: *“A strict parent with loving intentions”* is better than a parent allowing any behaviour “*out of apathy”* [CB]). Moral justification in the case study speaks to the power dynamic between humans and horses. The analogy of a strict parent used in the discourse we analysed is reminiscent of the "strict father” construct in political theory (Lakhoff 2016) wherein the parent is the moral guide and any deviation by the child must be punished. Justifications for whipping the horses in Case A and B certainly appear to fit this description.
2. Euphemistic labelling: using language to detract from harmful actions (*“Horses by nature thrive on direction given by a respected leader”* [CB]). Bandura called euphemising “an injurious weapon” (2002) as it has been used to make cruel and inhumane acts more socially acceptable. Regarding non-human animals, euphemistic language is used in animal agriculture to disguise practices within raising and slaughtering animals that might not be comfortable for society (Stibbe 2001; Croney & Reynnells 2008; Abrams *et al.* 2015). Similarly, the way in which harmful practices are sometimes termed “training” may allow individuals to disguise welfare-compromising actions.
3. Advantageous comparison: trivialising actions to make them seem less harmful (*“…she is only asking the horse to pick up its feet…not beating it”* [CA]). In this example, advantageous comparison allows one to redefine the act as a form of communication “to ask” a horse to do something rather than “beating” a horse in punishment or abuse. This does not consider the horse’s experience of the whip being used to produce the seemingly benign outcome.
4. Displacement of responsibility: shifting responsibility for actions to others (*“*[sport governing bodies need] *clearer guidelines, policies, and procedures concerning the best welfare for horses”* [CB]). Responsibility shifting has been observed in other animal welfare contexts such as laboratory animal use (Engel et al. 2020), but also previously in the horse world. For example, tight nosebands on horse bridles are used to increase control over the horse and reduce behavioural signs of discomfort that might be judged unfavourably in competition (Merkies *et al.* 2022). Despite evidence of the harm caused by tight nosebands, lack of rule enforcement is blamed for the issue (Visser *et al.* 2019). The need for governing bodies in equestrian sport to define, and enforce, acceptable practices support the idea that equestrians act as a collective culture.
5. Diffusion of responsibility: suggesting that there is no choice but to follow social norms (*‘The acceptable fix for this is to get the horse going forward in any way possible…”* [CB]). Mechanism 5 shows how practices are entangled with equestrian culture. The fact that welfare-compromising practices such as whipping exist as normalised practices, despite the availability of alternative training methods (Goodwin *et al.* 2009), is evidence of this.
6. Disregard or distortion of consequences: avoiding confrontation (*“It doesn’t amount to abuse to me, horse’s movements aren’t affected and I don’t see any injury being caused”* [CA]). In some cases, individuals chose to avoid judging the whipping or consider the subjective experience of the horses being whipped, which may reflect a disregard or distortion of consequences.
7. Dehumanisation: reducing perception of human qualities of the victims (*“Horses kick and bite each other all the time to signal to one another to move over, etc.* […] *you can observe that they communicate with each other by aggressive touching all the time”* [CA]). Although the focus of the current study was the welfare of horses, and thus the subjects logically incapable of being dehumanised, we consider a form of self-dehumanisation as a potential mechanism for moral disengagement here. In the case study, there were claims that the way the horses behave is a justification for a form of human behaviour towards another that would be considered unacceptable towards another human. This results in “self-dehumanisation”, which has been observed in many other contexts of immoral behaviour including social ostracising of others, online game violence, cheating and dishonesty (Bastian *et al.* 2012, 2013; Kouchaki *et al.* 2018). Self-dehumanisation functions to reduce the moral distress caused by one’s own immoral or unethical behaviour. In the present case, individuals may dehumanise their own actions to justify their behaviour towards horses. Therefore, if horses behave aggressively towards each other then it is justifiable for a human to do so as well, even if humans would not act this way towards each other.
8. Attribution of blame: blaming the victim for the harm inflicted (*“Some horses learn the easy way, and some just don’t. Horses are smart creatures, and they can learn to just refuse to do as they’re told…*[CB]). Victim blaming not only diminishes the subjective value of the one being harmed but also minimises the claim of the harm done. This is commonly seen in domestic and sexual abuse cases (Capezza & Arriaga 2008; Flynn *et al.* 2023). Blaming the victim also leads to reduced accountability for the harmful actions, which means the one committing the offence is less likely to change their behaviour in the future (Henning & Holdford 2006).

Moral disengagement theory provides one explanation for how and why individuals choose to diminish the welfare-comprising actions of some, even when wider society judges the acts as morally wrong. The mechanisms of moral disengagement may also provide targets for behaviour change strategies.

### Overall theme of polarisation

Contrasting views were expressed across all discourse topics analysed in this case study, in which discourse either supported the performance horse industry and practices depicted within, or was decidedly opposed. Some individuals justified, trivialised and reframed the use of tools like whips, bits and spurs, while others were firmly opposed to these. Horse-focused discourse either expressed some level of concern for the horse or placed blame on horse behaviour that required harsh treatment to correct. Perspectives on welfare were likewise strikingly varied, with some framing the events of Case A and B as “abuse” while others framed them as examples of poor or even acceptable horsemanship. The polarisation of perspectives overall interpreted in this study reveals perhaps the most important implication of this study: recognising tribalism can serve as both a barrier and opportunity for change.

Although tribalism is widely accepted as part of the social organisation of humans, it can create a barrier to cultural change (Greene 2013; Haidt 2020). The polarisation of views related to horses, equestrian activities and horse welfare as described above is evidence of the “us vs them” of tribalism. With discourse positioning individuals within their respective socio-moral tribes, there is no indication that other perspectives are given any real consideration. Furthermore, the apparent dismissal of the concerns of those who were so outraged by the actions in Case A and B may be a sort of “in-group policing”. This is a phenomenon described in polarised social group conflicts where the “in-group” attempts to silence or intimidate others who share conflicting views (Stanton 2013; Haidt 2020; Shissler 2025).

As discussed in a study on the motivation and maintenance of veganism, tribal bias reduces the likelihood of individuals engaging with out-groups (Cherry 2015). Vegans may be unlikely to engage with those attempting to dispute the benefits of veganism and meat-eaters may likewise not be open to information about veganism (Cherry 2015); however, engagement may result in a “backfire effect”, whereby individuals end up more strongly endorsing a misperception or controversial opinion (Abelson 1986; Nyhan & Reifler 2010). Resistance to change may be more likely or amplified when attempts to create change include insulting language (Hornsey & Esposo 2009), such as seen in this case study’s *ad hominem* discourse.

Not surprisingly then, when discourse in the social media groups was aimed at educating others, or where commentators attempted to argue their opposing positions by commenting on the perceived knowledge deficit of others, it was met with resistance. The knowledge deficit model assumes the attitudes and behaviours of others are underpinned by a simple lack of knowledge (Simis *et al.* 2016). Bail *et al.* (2018) and Merhout & Volfovsky (2018) likewise observed this effect in social media commentary where people shared political views and found that this only increased polarisation instead of opening minds to alternative views. If individuals are not already open to information, they are unlikely to engage constructively with it. This is similarly seen in science communication, where attempts at education are often perceived as judgement of the moral and practical norms of a group and have the opposite of the desired effect (Hart & Nisbett 2012; Simis *et al.* 2016).

Stemming from tribalism is the potential influence of collectivism (Bond 2004) vs individualism, which may be fundamental to the polarisation of the discourse described here. Collectivism is the tendency toward group interdependence and cohesion, whereas individualism is the focus on individual goals (Hofstede 1980). Social groups where individualism is valued as a group norm are more likely to behave individualistically (Jetten *et al.* 2002). In the context of the present case study, statements of belief from those attempting to promote animal welfare may be coming from an individualistic approach.

Although collectivism and individualism are culturally driven, there is evidence that social groups may also be subject to the effects of these attributes. Jetten *et al.* (2002) applied social identity theory and social categorisation theory to demonstrate this. Social identity theory indicates that individuals incorporate the norms of their social in-group into their personal identity (Tajfel 1982; Tajfel & Turner 1986), while social categorisation theory describes how humans tend to categorise each other based on social group membership (Turner *et al.* 1989). Our previously proposed idea that equestrians become enculturated and thereby uptake the attitudinal and practical norms of equestrian culture fits well within this social identity theory (Cheung *et al.* 2025). Social groups that have a strong social identity and deep-rooted collective social norms, such as equestrianism, are suggested to be more likely to have members that exhibit collectivist tendencies that guide their behaviour (Jetten *et al.* 2002). Those confirming the norms of equestrianism may be exhibiting a form of collectivism, which is strongly motivated to maintain social group norms (Tajfel 1982; Tajfel & Turner 1986). We observed this in *Discourse 2*, where the enculturated practices and attitudes of the equestrian industry may promote moral disengagement and protection of traditional views towards horses. We suggest that this finding underscores the need for the equestrian industry to re-ground its culture and practices in science-based evidence of horses and their welfare (McLean & McGreevy 2010; Uldahl & Mellor 2025).

Regardless of the legitimacy of the arguments that whipping constitutes abuse or poor horsemanship, people find moral judgement aversive and resist it (Minson & Monin 2012). Resistance to judgement of one’s moral self-identity may ultimately produce opposition to perspectives on horse welfare when arguments are presented in a way that leaves no room for discussion. Weiper and Vonk (2021) found that a less rigid approach to communicating with meat-eaters on moral standpoints on veganism and animal welfare was received with greater open-mindedness than when inflexible values were expressed. Ultimately, here we propose that strict arguments made by some in the social media comments we analysed may have the opposite of intended effects to help the horse world achieve progress in animal welfare. Neither argument for nor against whip use and other aversive training methods in the performance horse industry will gain traction if presented to equestrians simply as something that is deemed wrong, or to non-equestrians as a matter they just do not understand.

### Possible ways forward

Since there is a paucity of research on overcoming tribalised polarisation in animal welfare, we turn to work in the social psychology of political tribalism. Relevant proposed solutions to reduce the discord caused by tribalism are considered and applied to this case study.

One way of cooperating on polarised moral issues created by tribalism is proposed by Greene (2013), who advocates a utilitarian approach. Greene proposes a “meta-morality” that transcends tribalism (Greene 2013). This means finding a rational moral common ground where a solution is developed based on scientific evidence that provides the most benefit to the most individuals Greene 2013). If this solution can include the horse, i.e. improving horse welfare, then this approach might be advisable, though as evidenced throughout the case, what it means to care for horses and how human actions affect their welfare is variably interpreted and the weight given to human versus other animal interests may vary between interest-groups.

Another potential solution is offered by Haidt (2012), who encourages understanding the emotional position of others and interacting with outgroup members in what he terms “friendly” ways (2012). Furthermore, Haidt’s framework requires open-mindedness to the value of other moral viewpoints outside of one’s tribal group. This acceptance of moral pluralism is critiqued as there are moral tribes that include objectively harmful values, such as white supremacy groups (Clark & Bain-Selbo 2022), so this only works insofar as preservation of animal welfare is a shared value across groups. In the current context, there may be the opportunity to establish this as a guiding principle to which all can buy in, without initially defining it, or exploring related evidence.

Martha Nussbaum (2008, 2015) argues that the harmful aspects of tribalism are reduced, in part, through love and compassion. According to Nussbaum (2008, 2015), these emotions are a necessary guide for moral reasoning in place of the *post hoc* rationalisation often seen. She goes on to say that compassion and love expand our concerns to the well-being of all. This concept overlaps with some aspects of Greene’s utilitarian plan (2013) and Haidt’s consideration for other perspectives (2012). Perhaps, with compassion for the emotional experiences of others, constructive discussion could occur and polarisation of views on animal welfare might be reduced. Including horses (and other animals) in the circle of concern that Nussbaum indicates, by considering their needs as subjective beings in their own right, may further erode tribalistic speciesism.

### Study considerations

This instrumental case study sought to explore the phenomena of perceived performance horse welfare violations and how they are dealt with by people on social media. Social media as a data source has potential limitations associated with the selection of sites for data collection and the inability to identify participants. The social media environment creates potential biases in the scope of individuals commenting and may promote emotional responses (Morstatter & Liu 2017). We acknowledge that our specific findings are limited to the context of the social media discourse we analysed and not generalisable beyond this context, but our approach nonetheless gives insight into much wider issues relating to the social construction of animal welfare, which may inform other contexts.

Ethical considerations limited the identification of the case subjects and commentators on social media, which has prevented the potential to investigate underlying factors contributing to our findings at this time. However, we did not conduct this study with the intent of quantifying equestrian or non-equestrian perspectives, nor interpreting the meaning behind individuals’ comments. Rather we sought to identify discourse in relation to the case scenarios and understand their broader meaning based on the importation of relevant theories that are not widely considered within the field of animal welfare. This is an important distinction between other forms of qualitative research and qualitative discourse analysis.

## Animal welfare implications and conclusion

Others have considered individual motivations for the attitudes and behaviours that contribute to performance horse welfare issues (Luke *et al.* 2024; Mauricio *et al.* 2024). In addition, other work has included a “systems thinking” approach designed to encompass the culture of equestrianism and all those within it (Ross *et al.* 2025). With this case study, we expand on these works with novel attention given to the broader socio-moral construction of perspectives on animals as beings, how we treat them, and what welfare for them means. This highlights the importance of contributions from human sociology and social psychology to understand the attitudes and morals that influence how we care for and about animals.

To the authors’ knowledge, discussion of performance equestrian culture in light of tribalism and subsequent moral disengagement has not been done previously. This work also highlights the wider value of consideration of the role of tribalism in the socio-moral co-creation of animal welfare perspectives and the resulting polarisation of views, which has important practical implications for bringing about change. These findings might be built upon to encourage positive change for animal welfare by compassionately meeting individuals at their position on animal welfare (and the lines they draw between acceptable and unacceptable practice) and opening constructive discussion to break down barriers created by tribal tendencies.

## References

[r1] Abelson RP 1986 Beliefs are like possessions. Journal for the Theory of Social Behaviour 16(3): 223–250. 10.1111/j.1468-5914.1986.tb00078.x

[r2] Abrams D, De Randsley Moura G and Travaglino GA 2013 A double standard when group members behave badly: Transgression credit to ingroup leaders. Journal of Personality and Social Psychology 105(5): 799–815. 10.1037/a0033600 23895265

[r3] Abrams KM, Zimbres T and Carr C 2015 Communicating sensitive scientific issues: The interplay between values, attitudes, and euphemisms in communicating livestock slaughter. Science Communication 37(4): 485–505. 10.1177/1075547015588599

[r4] Anthony M 2020 Web Wide Warfare. Part 1: The Blue Shadow. Journal of Futures Studies 24(4) 35–50 10.6531/JFS.202003 24(3).0002

[r140] Armstrong, G. 2003. Football hooligans: Knowing the score (Paperback ed., reprinted). Berg. Oxford, UK.

[r141] Ashokkumar, A., Galaif, M., & Swann, W. B. 2019. Tribalism can corrupt: Why people denounce or protect immoral group members. Journal of Experimental Social Psychology, 85, 103874. 10.1016/j.jesp.2019.103874

[r142] Aquino, K., & Reed, A. 2002. The self-importance of moral identity. Journal of Personality and Social Psychology, 83(6), 1423–1440. 10.1037/0022-3514.83.6.1423 12500822

[r5] Bail CA, Argyle LP, Brown TW, Bumpus JP, Chen H, Hunzaker MBF, Lee J, Mann M, Merhout F and Volfovsky A 2018 Exposure to opposing views on social media can increase political polarization. Proceedings of the National Academy of Sciences 115(37): 9216–9221. 10.1073/pnas.1804840115 PMC6140520 30154168

[r8] Bandura A 1990 Selective activation and disengagement of moral control. Journal of Social Issues 46(1): 27–46. 10.1111/j.1540-4560.1990.tb00270.x

[r9] Bandura A 2002 Selective moral disengagement in the exercise of moral agency. Journal of Moral Education 31(2): 101–119. 10.1080/0305724022014322

[r11] Bastian B, Jetten J and Radke HR 2012 Cyber-dehumanization: Violent video game play diminishes our humanity. Journal of Experimental Social Psychology 48(2): 486–491. 10.1016/j.jesp.2011.10.009

[r12] Bastian B, Jetten J, Chen H, Radke HR, Harding JF and Fasoli F 2013 Losing our humanity: The self-dehumanizing consequences of social ostracism. Personality and Social Psychology Bulletin 39(2): 156–169. 10.1177/0146167212471205 23386654

[r13] Bastian B and Loughnan S 2017 Resolving the meat-paradox: A motivational account of morally troublesome behavior and its maintenance. Personality and Social Psychology Review 21(3), 278–299. 10.1177/1088868316647562 27207840

[r14] Berger PL and Luckmann T 2011 The social construction of reality: A treatise in the sociology of knowledge. Open Road Media Integrated Media: New York, NY, USA.

[r15] Bond R 2004 Conformity across cultures. Encyclopedia of Applied Psychology 1: 457. Academic Press, New York.

[r16] Borovali M 2018 Ad hominem argumentation in politics. Philosophy & Social Criticism 44(4): 426–436. 10.1177/0191453718755206

[r18] Bouvier G 2015 What is a discourse approach to Twitter, Facebook, YouTube and other social media: Connecting with other academic fields? Journal of Multicultural Discourses 10(2): 149–162. 10.1080/17447143.2015.1042381

[r19] Boyd R and Richerson PJ 2005 The origin and evolution of cultures. Oxford University Press: London, UK.

[r20] Brady WJ, Crockett MJ and Van Bavel JJ 2020 The MAD model of moral contagion: The role of motivation, attention, and design in the spread of moralized content online. Perspectives on Psychological Science 15(4): 978–1010. 10.1177/1745691620917336 32511060

[r21] Bradley C and Roland J 2022 Critical Thinkers for a Critical Time: Debate as a Foundation for Youth Civic Engagement. National Civic Review 111(2): 14–22. https://www.jstor.org/stable/48680128 (accessed 15 May 2026).

[r22] Braun V and Clarke V 2022 Conceptual and design thinking for thematic analysis. Qualitative Psychology 9(1): 3. 10.1037/qup0000196

[r24] Burr V and Dick P 2017 Social constructionism. The Palgrave Handbook of Critical Social Psychology (pp 59–80). Palgrave Macmillan: London, UK.

[r25] Capezza NM and Arriaga XB 2008 Why do people blame victims of abuse? The role of stereotypes of women on perceptions of blame. Sex Roles 59(11–12): 839–850. 10.1007/s11199-008-9488-1

[r27] Cherry E 2015 I was a teenage vegan: Motivation and maintenance of lifestyle movements. Sociological Inquiry 85(1): 55–74. 10.1111/soin.12061

[r28] Cheung E, Mills D and Ventura BA 2025 ‘But my horse is well cared for’: A qualitative exploration of cognitive dissonance and enculturation in equestrian attitudes toward performance horses and their welfare. Animal Welfare 34(50). 10.1017/awf.2025.10028 PMC12304784 40735428

[r30] Chung W and Zeng D 2020 Dissecting emotion and user influence in social media communities: An interaction modeling approach. Information & Management 57(1): 103108. 10.1016/j.im.2018.09.008

[r31] Clark KM and Bain-Selbo E 2022 Tribalism and compassion in the age of a pandemic. Soundings: An Interdisciplinary Journal 105(2): 143–223. 10.5325/soundings.105.2.0143

[r32] Clark CJ, Liu BS, Winegard BM and Ditto PH 2019 Tribalism is human nature. Current Directions in Psychological Science 28(6): 587–592.

[r34] Costello K and Hodson G 2010 Exploring the roots of dehumanization: The role of animal–human similarity in promoting immigrant humanization. Group Processes & Intergroup Relations 13(1): 3–22. 10.1177/1368430209347725

[r35] Cova B and Cova V 2001 Tribal aspects of postmodern consumption research: The case of French in-line roller skaters. Journal of Consumer Behaviour 1(1): 67–76. 10.1002/cb.54

[r36] Cova B and Cova V 2002 Tribal marketing: The tribalisation of society and its impact on the conduct of marketing. European Journal of Marketing 36(5/6): 595–620. 10.1108/03090560210423023

[r37] Cowen T 2006 Market failure for the treatment of animals. Society 43(2): 39–44. 10.1007/BF02687369

[r38] Creswell JW and Poth CN 2016 Qualitative Inquiry and Research Design: Choosing Among Five Approaches. Sage Publications: Los Angeles, CA, USA.

[r39] Croney CC and Reynnells RD 2008 The ethics of semantics: Do we clarify or obfuscate reality to influence perceptions of farm animal production? Poultry Science 87(2): 387–391. 10.3382/ps.2007-00404 18212386

[r40] Darwin C 1871/1981 The Descent of Man and Selection in Relation to Sex. Princeton University: Princeton, USA.

[r41] Denzin NK and Lincoln YS 2011 The Sage Handbook of Qualitative Research. Sage: London, UK.

[r42] Ekman M 2019 Anti-immigration and racist discourse in social media. European Journal of Communication 34(6): 606–618.

[r43] Engel SM and Philosophy Documentation Center 1994 The five forms of the ad hominem fallacy: Inquiry: Critical Thinking Across the Disciplines 14(1): 19–36. 10.5840/inquiryctnews199414123

[r44] Engel RM, Silver CC, Veeder CL and Banks RE 2020 Cognitive dissonance in laboratory animal medicine and implications for animal welfare. Journal of the American Association for Laboratory Animal Science 59(2): 132–138. 10.30802/AALAS-JAALAS-19-000073 31918791 PMC7073402

[r45] Eriksson P and Kovalainen A 2008 Qualitative Methods in Business Research. Sage: London, UK.

[r46] Espinosa R and Treich N 2024 Animal welfare as a public good. Ecological Economics 216: 108025. 10.1016/j.ecolecon.2023.108025

[r47] Ferrera SJ 2015 Tribalism: biological roots and emotional process. Family Systems: A Journal of Natural Systems Thinking in Psychiatry & the Sciences 11(1).

[r48] Flynn A, Cama E, Powell A and Scott AJ 2023 Victim-blaming and image-based sexual abuse. Journal of Criminology 56(1): 7–25. 10.1177/26338076221135327

[r49] Fraser D, Weary DM, Pajor EA and Milligan BN 1997 A scientific conception of animal welfare that reflects ethical concerns. Animal Welfare 6(3): 187–205. 10.1017/S0962728600019795

[r50] Gee JP 2014 An Introduction to Discourse Analysis: Theory and Method, Fourth Edition. Routledge: London, UK.

[r51] Gilbert I, Poole R and Niemi L 2025 The sociomoral construction of the dairy industry on social media. Qualitative Research in Psychology 22(1): 286–313. 10.1080/14780887.2024.2319760

[r52] Gill R 2000 Discourse analysis. *Qualitative researching with text*, image and sound 1: 172–190. 10.4135/9781849209731.n10

[r53] Goodwin D, McGreevy P, Waran N and McLean A 2009 How equitation science can elucidate and refine horsemanship techniques. The Veterinary Journal 181(1): 5–11. 10.1016/j.tvjl.2009.03.023 19394880

[r54] Greene J 2013 Moral tribes: Emotion, reason, and the gap between us and them. Penguin: New York, NY, USA.

[r55] Grefe G and Pickel-Chevalier S 2015 The equine business: the spectacular growth of a new equine segment market in France. The New Equine Economy in the 21st Century. *Wageningen Academic Publishers*: Wageningen, The Netherlands.

[r56] Guba EG and Lincoln YS 1994 Competing paradigms in qualitative research. Handbook of Qualitative Research 2(163–194): 105.

[r57] Haidt J 2012 The Righteous Mind: Why Good People are Divided by Politics and Religion. Pantheon/Random House: New York, USA.

[r58] Haidt J 2020 Tribalism, Forbidden Baserates, and the Telos of Social Science. Psychological Inquiry 31(1): 53–56. 10.1080/1047840X.2020.1722602

[r59] Hart PS and Nisbet EC 2012 Boomerang effects in science communication: How motivated reasoning and identity cues amplify opinion polarization about climate mitigation policies. Communication Research 39(6): 701–723. 10.1177/0093650211416646

[r60] Henning K and Holdford R 2006 Minimization, denial, and victim blaming by batterers: How much does the truth matter? Criminal Justice and Behavior 33(1): 110–130. 10.1177/0093854805282322

[r61] Herskovits MJ 1949 Man and his works; the science of cultural anthropology. Alfred A Knopf: New York, USA.

[r62] Hofstede G 1980 Culture’s consequences: International differences in work-related values. Sage: Beverly Hills, CA, USA.

[r63] Hornsey MJ and Esposo S 2009 Resistance to group criticism and recommendations for change: Lessons from the intergroup sensitivity effect. Social and Personality Psychology Compass 3: 275–291. 10.1111/j.1751-9004.2009.00178.x

[r64] Hull K, Warren C and Smith K 2024 Politics makes bastards of us all: Why moral judgment is politically situational. Political Psychology 45(6): 1013–1029. 10.1111/pops.12954

[r65] Hyvärinen H and Beck R 2018 Emotions trump facts: The role of emotions in on social media: A literature review. Proceedings of the Annual Hawaii International Conference on System Sciences (pp 1797–1806). IEEE Computer Society Press: USA.

[r66] Jaquet F 2022 Speciesism and tribalism: Embarrassing origins. Philosophical Studies 179(3): 933–954. 10.1007/s11098-021-01700-6

[r67] Jetten J, Postmes T and McAuliffe BJ 2002 ‘We’re all individuals’: Group norms of individualism and collectivism, levels of identification and identity threat. European Journal of Social Psychology 32(2): 189–207. 10.1002/ejsp.65

[r68] Jerit J 2008 Issue framing and engagement: Rhetorical strategy in public policy debates. Political Behavior 30(1): 1–24. 10.1007/s11109-007-9041-x

[r69] Johansson-Stenman O 2018 Animal welfare and social decisions: Is it time to take Bentham seriously? Ecological Economics 145: 90–103. 10.1016/j.ecolecon.2017.08.019

[r71] Joy M 2009 Why we Love Dogs, Eat Pigs, and Wear Cows: An Introduction to Carnism. Conari Press: San Francisco, CA, USA.

[r72] Kelly DR 2013 24 Moral disgust and the tribal instincts hypothesis. Cooperation and its evolution (pp 503). MIT Press: USA.

[r73] Kouchaki M, Dobson KSH, Waytz A and Kteily NS 2018 The link between self-dehumanization and immoral behavior. Psychological Science 29(8): 1234–1246. 10.1177/0956797618760784 29787345

[r74] Krzyżanowski M and Tucker JA 2018 Re/constructing politics through social and online media: Discourses, ideologies, and mediated political practices. Journal of Language and Politics 17(2): 141–154.

[r75] Lakoff G 2016 Moral Politics: How Liberals and Conservatives Think. University of Chicago Press: Chicago, USA.

[r76] Loughnan S and Davies T 2020 The meat paradox. Why we Love and Exploit Animals (pp 171–187). Routledge: London, UK.

[r77] Luke KL, Rawluk A, McAdie T, Smith BP and Warren-Smith AK 2024 Investigating the motivational priorities underlying equestrians’ horse-keeping and training practices. Anthrozoös 37(3): 479–499. 10.1080/08927936.2024.2303228

[r78] Lusk JL and Norwood FB 2008 A survey to determine public opinion about the ethics and governance of farm animal welfare. Journal of the American Veterinary Medical Association 233(7): 1121–1126. 10.2460/javma.233.7.1121 18828724

[r79] Lusk JL and Norwood FB 2011 Animal welfare economics. Applied Economic Perspectives and Policy 33(4): 463–483.

[r80] Macagno F 2013 Strategies of character attack. Argumentation 27(4): 369–401. 10.1007/s10503-013-9291-1

[r81] Machery E 2016 The evolution of tribalism. In: Kiverstein J (ed) The Routledge Handbook of Philosophy of the Social Mind (pp 104–117). Routledge: London, UK.

[r82] Maffesoli M and Maffesoli M 1996 The time of the tribes: The decline of individualism in mass society (Online-Ausg). SAGE: London, UK.

[r83] Maréchal G 2010 Encyclopedia of Case Study Research. Sage: London, UK.

[r84] Maurício LS, Leme DP and Hötzel MJ 2024 The easiest becomes the rule: Beliefs, knowledge and attitudes of equine practitioners and enthusiasts regarding horse welfare. Animals 14(9): 1282. 10.3390/ani14091282 38731286 PMC11083815

[r85] Mead M 1963 Papers in honor of Melville J. Herskovits: Socialization and enculturation. Current Anthropology 4(2): 184–188. https://www.jstor.org/stable/2739841 (accessed 30 June 2025).

[r86] Mellor DJ, Beausoleil NJ, Littlewood KE, McLean AN, McGreevy PD, Jones B and Wilkins C 2020 The 2020 five domains model: Including human–animal interactions in assessments of animal welfare. Animals 10(10): 1870. 10.3390/ani10101870 33066335 PMC7602120

[r87] Merhout F and Volfovsky A 2018. Exposure to opposing views on social media can increase political polarization. Proceedings of the National Academy of Sciences 115(37): 9216–9221. 10.1073/pnas.1804840115 PMC6140520 30154168

[r88] McLean AN and McGreevy PD 2010 Ethical equitation: Capping the price horses pay for human glory. Journal of Veterinary Behavior 5(4): 203–209. 10.1016/j.jveb.2010.04.003

[r89] Merkies K, Copelin C, Small N and Young J 2022 Noseband fit: Measurements and perceptions of Canadian equestrians. Animals 12(19): 2685. 10.3390/ani12192685 36230425 PMC9559623

[r90] Mills DS 2025 A psychobiological framework for defining discrete emotions in animals. Applied Animal Behaviour Science 286: 106595. 10.1016/j.applanim.2025.106595

[r91] Minson JA and Monin B 2012 Do-gooder derogation: Disparaging morally motivated minorities to defuse anticipated reproach. Social Psychological and Personality Science 3(2): 200–207. 10.1177/1948550611415695

[r93] Moore C 2015 Moral disengagement. Current Opinion in Psychology 6: 199–204. 10.1016/j.copsyc.2015.07.018

[r95] Morstatter F and Liu H 2017 Discovering, assessing, and mitigating data bias in social media. Online Social Networks and Media 1: 1–13. 10.1016/j.osnem.2017.01.001

[r96] Muhammad M, Stokes JE, Morgans L and Manning L 2022 The social construction of narratives and arguments in animal welfare discourse and debate. Animals 12(19): 2582. 10.3390/ani12192582 36230322 PMC9559530

[r98] Nussbaum MC 2008 Upheavals of Thought: The intelligence of Emotions, Eighth Printing. Cambridge University Press: Cambridge, UK.

[r99] Nussbaum MC 2015 Political Emotions: Why Love Matters for Justice, First Paperback Edition. Belknap Press of Harvard University Press: USA.

[r100] Nyhan B and Reifler J 2010 When corrections fail: The persistence of political misperceptions. Political Behavior 32(2): 303–330. 10.1007/s11109-010-9112-2

[r101] Phillips L and Jorgensen M 2002 Critical discourse analysis. *Discourse Analysis: As Theory and Method*. Sage Publications Limited: New York, USA.

[r102] Piazza J, Ruby MB, Loughnan S, Luong M, Kulik J, Watkins HM and Seigerman M 2015 Rationalizing meat consumption. The 4Ns. Appetite 91:114–128. 10.1016/j.appet.2015.04.011 25865663

[r103] Potter J and Wetherell M 2002 Analyzing discourse. In Analysing Qualitative Data. Routledge, London, UK, pp. 61–80.

[r105] Riggio G, Angori E, Menchetti L and Diverio S 2023 The link between the perception of animal welfare and the emotional response to pictures of farm animals kept in intensive and extensive husbandry systems: An Italian survey. Veterinary Sciences 10(11): 652. 10.3390/vetsci10110652 37999475 PMC10675316

[r106] Ross M, Proudfoot K, Merkies K, Lundgren C and Ritter C 2025 A wicked problem: Systemic issues surrounding Canadian equestrian dressage and dressage horse welfare. Animal Welfare 34: e11. 10.1017/awf.2025.2 39935780 PMC11810510

[r107] Roth W 2001 ‘Enculturation’: Acquisition of conceptual blind spots and epistemological prejudices. British Educational Research Journal 27(1): 5–27. 10.1080/01411920123822

[r108] Rothgerber H 2020 Meat-related cognitive dissonance: A conceptual framework for understanding how meat eaters reduce negative arousal from eating animals. Appetite 146 104511. 10.1016/j.appet.2019.104511 31707073

[r109] Rozin P, Lowery L, Imada S and Haidt J 1999 The CAD triad hypothesis: A mapping between three moral emotions (Contempt, anger, disgust) and three moral codes (Community, autonomy, divinity). Journal of Personality and Social Psychology 76(4): 574–586. 10.1037/0022-3514.76.4.574 10234846

[r112] Schüßler C, Nicolai S, Stoll-Kleemann S and Bartkowski B 2024 Moral disengagement in the media discourses on meat and dairy production systems. Appetite 196: 107269. 10.1016/j.appet.2024.107269 38360400

[r113] Searle JR 1995 The Construction of Social Reality. Simon and Schuster: NewYork, USA.

[r114] Shimahara N 1970 Enculturation-A reconsideration. Current Anthropology 11(2): 143–154. https://www.jstor.org/stable/2740527 (accessed 15 May 2026).

[r115] Simis MJ, Madden H, Cacciatore MA and Yeo SK 2016 The lure of rationality: Why does the deficit model persist in science communication? Public Understanding of Science 25(4): 400–414. 10.1177/0963662516629749 27117768

[r116] Singer P 2002 Animal Liberation. Ecco: London, UK.

[r117] Stake RE 1995 The Art of Case Study Research. Sage Publications: London, UK.

[r118] Stake RE 2005 Qualitative Case Studies. In: Denzin NK and Lincoln YS (eds) The Sage Handbook of Qualitative Research, Third Edition (pp 443–466). Sage Publications Ltd: London, UK.

[r119] Stangor C 2020 Exploring attitudes. In: Jhangiani R and Tarry H (eds) *Principles of Social Psychology-1st International Edition.* BC Campus: Victoria, BC, Canada. https://opentextbc.ca/socialpsychology (accessed 15 May 2026).

[r120] Stanton JA 2013 Terrorism in the context of civil war. The Journal of Politics 75(4): 1009–1022. 10.1017/S0022381613000984

[r121] Stibbe A 2001 Language, power and the social construction of animals. Society & Animals 9(2): 145–161. 10.1163/156853001753639251

[r122] Tajfel H 1982 Social Identity and Intergroup Relations. Cambridge University Press: Cambridge, UK.

[r123] Tajfel H and Turner JC 1986 The social identity theory of intergroup behaviour. Psychology of Intergroup Relations 2: 7–24.

[r124] Tetlock PE, Kristel OV, Elson SB, Green MC and Lerner JS 2000 The psychology of the unthinkable: Taboo trade-offs, forbidden base rates, and heretical counterfactuals. Journal of Personality and Social Psychology 78(5): 853–870. 10.1037/0022-3514.78.5.853 10821194

[r125] Travaglino GA, Abrams D, Randsley De Moura G and Yetkili O 2016 Fewer but better: Proportionate size of the group affects evaluation of transgressive leaders. British Journal of Social Psychology 55(2): 318–336. 10.1111/bjso.12125 26334165

[r126] Tucker JA, Theocharis Y, Roberts ME and Barberá P 2017 From liberation to turmoil: Social media and democracy. Journal of Democracy 28(4): 46–59. https://muse.jhu.edu/pub/1/article/671987 (accessed 15 May 2026).

[r127] Turner JC, Wetherell MS and Hogg MA 1989 Referent informational influence and group polarization. British Journal of Social Psychology 28: 135–147. 10.1111/j.2044-8309.1989.tb00855.x

[r128] Uldahl M and Mellor DJ 2025 Regulatory Integrity and Welfare in Horse Sport: A Constructively Critical Perspective. Animals 15(13): 1934. 10.3390/ani15131934 40646833 PMC12248743

[r129] Van Dijk TA 1997 The study of discourse. Discourse as Structure and Process 1(34): 703–752.

[r130] Van Eemeren FH, Garssen B and Meuffels B 2012 The disguised abusive ad hominem empirically investigated: Strategic manoeuvring with direct personal attacks. Thinking & Reasoning 18(3): 344–364. 10.1080/13546783.2012.678666

[r131] Visser EK, Kuypers MM, Stam JS and Riedstra B 2019 Practice of noseband use and intentions towards behavioural change in Dutch equestrians. Animals 9(12): 1131. 10.3390/ani9121131 31842468 PMC6940946

[r132] Walton DN 1998 Ad hominem arguments. University of Alabama Press: USA.

[r133] Wahl-Jorgensen K 2018 The emotional architecture of social media. *A Networked Self and Platforms, Stories, Connections* (pp 77–93). Routledge: London, UK.

[r134] Weary D and Robbins J 2019 Understanding the multiple conceptions of animal welfare. Animal Welfare 28(1): 33–40. 10.7120/09627286.28.1.033

[r135] Weiper ML and Vonk R 2021 A communicational approach to enhance open-mindedness towards meat-refusers. Appetite 167: 105602. 10.1016/j.appet.2021.105602 34284066

[r136] Wilson EO 2012 The social conquest of earth. WW Norton & Company, Liveright Publishing Corporation, London, UK.

[r137] **World Horse Welfare** 2024 *Review of the Use of the Whip in Racing.* https://www.worldhorsewelfare.org/what-we-do/sport-and-leisure-horses/our-work-with-regulators/review-of-the-use-of-the-whip-in-racing (accessed on 12 October 2025).

[r138] Yin RK 2003 Designing case studies. Qualitative Research Methods 5(14): 359–386.

[r139] Youniss J 1981 Moral development through a theory of social construction: An analysis. Merrill-Palmer Quarterly 27(4): 385–403

